# Epidemiologic study of drowning in Mashhad during 2014-2017 

**Published:** 2019-07

**Authors:** Golnaz Peyravi, Mohammadreza Joya, Reza Vafaeinezhad

**Affiliations:** ^*a*^Department of Risk and Disaster Management, Deputy of Health, Mashhad University of Medical Sciences, Mashhad, Iran.

## Abstract

**Background:**

As the summer approaches, many people go for swimming but sometimes this pleasurable activity has no happy ending. Drowning is an important health problem but often neglected. It is defined as choking (breathing problems) as a result of immersion in a fluid. Every summer, media publish reports of deaths due to drowning and warn people about it. However, many people recognize the risk of drowning only on the populous beaches not wherever close to water, like a bathtub or a large water puddle in the courtyard corner. The world total deaths due to drowning is estimated to be 6.8/100,000 people which is the second common cause of unintentional mortalities after road accidents. Most drownings occur in provincial beaches, but other parts of the country account for a significant proportion of injuries and deaths due to drowning/choking in water. This study aimed at investigating the epidemiologic aspects of drownings in Mashhad during 2014-2017.

**Methods:**

In this cross-sectional study, the data from the epidemiological survey of drowning cases during 2014-2017 were analyzed using SPSS software.

**Results:**

The total number of drownings reported to the Emergency Department during this period was 68. Most of which occurred in 2015 (35%), 26% of cases occurred in urban and 74% in rural areas. 75% were men and 25% women. 19% of deaths associated with drowning. 15% of drowning events occurred in recreational sport centers. Most cases were under 5 years old (45%), followed by adolescents and young groups (35%).

**Conclusions:**

Most of the drowning victims were children. Therefore, it is essential for parents to protect their children close to water, especially in private areas such as pools in gardens. We should make such places safe. Special attention should also be given to safety equipment, protection and training in public swimming pools and recreational facilities.

**Keywords:**

Drowning, Mashhad, Cross-sectional

